# Variable effect of co-infection on the HIV infectivity: Within-host dynamics and epidemiological significance

**DOI:** 10.1186/1742-4682-9-9

**Published:** 2012-03-19

**Authors:** Diego F Cuadros, Gisela García-Ramos

**Affiliations:** 1 Department of Biology, University of Kentucky, Lexington, KY, USA; 2 Infectious Disease Epidemiology Group, Weill Cornell Medical College, Doha, Qatar

## Abstract

**Background:**

Recent studies have implicated viral characteristics in accounting for the variation in the HIV set-point viral load (spVL) observed among individuals. These studies have suggested that the spVL might be a heritable factor. The spVL, however, is not in an absolute equilibrium state; it is frequently perturbed by immune activations generated by co-infections, resulting in a significant amplification of the HIV viral load (VL). Here, we postulated that if the HIV replication capacity were an important determinant of the spVL, it would also determine the effect of co-infection on the VL. Then, we hypothesized that viral factors contribute to the variation of the effect of co-infection and introduce variation among individuals.

**Methods:**

We developed a within-host deterministic differential equation model to describe the dynamics of HIV and malaria infections, and evaluated the effect of variations in the viral replicative capacity on the VL burden generated by co-infection. These variations were then evaluated at population level by implementing a between-host model in which the relationship between VL and the probability of HIV transmission per sexual contact was used as the within-host and between-host interface.

**Results:**

Our within-host results indicated that the combination of parameters generating low spVL were unable to produce a substantial increase in the VL in response to co-infection. Conversely, larger spVL were associated with substantially larger increments in the VL. In accordance, the between-host model indicated that co-infection had a negligible impact in populations where the virus had low replicative capacity, reflected in low spVL. Similarly, the impact of co-infection increased as the spVL of the population increased.

**Conclusion:**

Our results indicated that variations in the viral replicative capacity would influence the effect of co-infection on the VL. Therefore, viral factors could play an important role driving several virus-related processes such as the increment of the VL induced by co-infections. These results raise the possibility that biological differences could alter the effect of co-infection and underscore the importance of identifying these factors for the implementation of control interventions focused on co-infection.

## Background

At present, the role of viral genetic factors in the HIV epidemic is poorly understood. Many different HIV genetic subtypes circulate worldwide as well as their recombinant forms [1]. Moreover, there is a substantial variation in the length of the asymptomatic (chronic) phase of HIV infection, ranging from a few months to many years. This variation has been correlated with the so-called set point viral load (spVL), defined as the measure of the HIV concentration in blood during the chronic stage of the infection, representing the quasi-steady state equilibrium between virus production and clearance [1,2].

The functional differences and epidemiological significance of these variations are still unclear [3,4]. Several studies have indicated that host genetic factors can partially explain the variation observed in the spVL [5-7]. In addition, some other studies have revealed potential differences in the replicative capacity among different virus genotypes [8-13]. As a result, it has been suggested that if viral factors can affect the efficiency of viral replication and can be preserved from one infection to the next, this would imply a significant role of viral genetic factors determining the spVL [14].

Recent studies conducted to assess this hypothesis have evidenced that virus characteristics might indeed explain a significant proportion of the variation in the viral load (VL) observed among individuals and suggested that the spVL might be a heritable factor [15-18]. Further studies examining the HIV replicative capacity support this association between the replicative capacity of the virus and the VL [19,20]. These results have profound implications for important virus-related processes in which the VL is a key determinant, such as the progression, and especially in the transmission of the infection. This is given that infectiousness can be directly correlated with the concentration of HIV-RNA in plasma, which associates with shedding of the virus into genital track secretions [21,22]. The evidence suggests a strong correlation between the VL and HIV transmission rates [23-25]. Consequently, if viral genetic factors influence the concentration of the virus during the chronic stage of the infection, these viral characteristics might indeed play an important role on the transmission of the infection.

The spVL, however, is not in an absolute equilibrium state; it is frequently perturbed by immune activations generated by the invasion of other pathogens, resulting in a significant amplification of the VL. Changes in the host immune response induced by concomitant infections (co-infection) may account for transient increments in the VL that could make the host more infectious and increase the risk of transmission of the virus during the chronic stage of the infection [26-28]. This transient amplification of the VL generated by co-infection may be an important contributor to the successful transmission of the infection, especially in areas were the immune system is constantly challenged by multiple infections, such as in sub-Saharan Africa [29-31].

Furthermore, there is substantial heterogeneity in the effect of co-infection at the individual and population levels. Within individuals, the co-infection induced increment of the VL varies greatly [32-34]. Likewise, the effect of co-infection in the spread of the infection at the population level depends on several behavioral and epidemiological factors related to the stage and the distribution of the HIV epidemic within the population. Co-infections with sexually transmitted infections (STI) such as gonorrhea and chlamydia would have the highest impact on the transmission of the virus in populations where the epidemic is concentrated in the high risk (core) groups [35,36]. Conversely, infections prevalent in the general population such as malaria and herpes simplex virus type 2 (HSV-2) would be important contributors to the spread of the infection when the epidemic has invaded the general population [36,37].

Based on the biological evidence previously discussed, we suspect that viral factors might contribute to the variation in the effect of co-infection at host and population levels. Here, taking a simple modeling approach, we examined how viral factors might alter the dynamics of the virus population in the presence of co-infection and generated variation in the effects of co-infection among individuals. We expect that viruses with high replicative capacity that sustain elevated spVL would also generate larger transient increments in the VL in response to host immune activations induced by co-infection. In contrast, viruses with poor capacity to infect target cells and replicate would generate lower spVL and consequently lower VL increments in response of co-infection. Since the transmission efficiency of the virus among individuals is associated with the viral burden, highly infectious individuals would have viral characteristics that maximize the increment of the VL in the presence of co-infection. This result might have important implications at both the individual and the population levels.

To assess this hypothesis, we generated a mathematical model that describes the dynamics of HIV and a concomitant pathogen within the host to evaluate the variation of the viral replicative capacity on the spVL and the increment on the VL in response to co-infection. Additionally, we evaluated the effects of these variations at the population level.

## Methods

We developed a deterministic differential equation compartmental model based on two independent models that describe the dynamics of HIV and a parasitic infection, namely malaria.

### HIV model

The basic model of HIV dynamics implemented here has three variables: *T *the population sizes of uninfected cells, T* infected cells, and *V *free virus particles [38-40]. These quantities denote the total abundance of the given population per milliliter (mL) of blood, and their dynamic is described by the following system of differential equations:

(1) $$\frac{dT}{dt}=s-\left(d_{t}+k_{v}V\right)T$$

(2) $$\frac{dT^{*}}{dt}=k_{v}VT-\delta T^{*}$$

(3) $$\frac{dV}{dt}=N\delta T^{*}-cV,$$

where *s *represents the production rate of activated immune (CD4+ T) cells, *d_t _*the natural death rate of activated immune cells, *k_v _*the HIV infection rate of activated immune cells, *δ *death rate of HIV infected cells, *N *the HIV production rate by an infected cell, and *c *the HIV removal rate. The magnitudes of these parameters are summarized in Table 1. From this system of differential equations it is possible to determine the concentration of the virus population at equilibrium, which might be associated with the spVL in the chronic stage of the infection, and is described by the following equation [38]:

**Table 1:** Co-infection within-host model parameter values

Parameter	Value	Reference
Rate of red blood cell production (Δ)	2.5 × 10^8 ^cells/ml day	[49,50,79]

Natural death rate of uninfected red blood cells (*μ_x_*)	0.0083/day	[46,49]

Infection rate of red blood cells by merozoites (*k_m_*)	2.5 × 10^-10^/merozoite day	[46,49]

Differentiation rate of merozoite (*μ_1_*)	0.5/day	[46,49]

Natural death rate of infected blood cells (*μ_y_*)	0.025/day	[46,49]

Clearance rate of infected red blood cells due to the immune system (*μ_c_*)	1 × 10^-8^/cell day	[50]

Natural death rate of free merozoites (*μ_m_*)	48/day	[46,49]

Death rate of merozoites by contact with immune cells (*μ_h_*)	1 × 10^-8^/cell day	[46,49]

Merozoite production per infected red blood cell (*r*)	16	[46,49]

Production rate of immune cells (malaria specific) (*ε*)	1 × 10^-4 ^cell/ml day	[47]

Proliferation rate of immune cells in response to infected red blood cells (*λ_y_*)	2 × 10^-8^/cell day	[50]

Proliferation rate of immune cells in response to merozoites (*λ_m_*)	3 × 10^-8^/merozoite day	[50]

Maximum number of activated immune cells per ml of blood (*T_max_*)	1.5 × 10^6 ^cell/ml	[58]

HIV infection rate for nonspecific immune cell (*k_v_*)	[0.1 × 10^-5 ^- 2 × 10^-5^] virion/day	Representative values

Production rate of immune cells (*s*)	1 × 10^4 ^cell/ml day	[38]

Growth rate of non-specific immune cells (*r_T_*)	0.03/day	[80]

Natural death rate of immune cells (*d_t_*)	0.01/day	[81]

Death rate of HIV infected cells (*δ*)	0.7/day	[58]

HIV production by an infected cell (*N*)	[20-300] virions/cell	Representative values

HIV removal rate (*c*)	23/day	[82]

(4) $$\bar{V}=\frac{Ns}{\delta c}-\frac{d_{t}}{k_{v}}.$$

This equation shows that virus concentration at equilibrium ($\bar{V}$) may depend on virus factors that determine its replicative capacity: the infection rate of immune cells (*k_v_*) and the production rate by infected cells (*N*). Since these parameters represent intrinsic characteristics of the virus, we focused on evaluating the effect of such variations on the VL burden generated by the presence of a concomitant pathogen.

### Malaria model

Malaria was selected as the concomitant infection based on substantial biological and epidemiological evidence suggesting an important malaria-HIV interaction at the individual level and across regions where their incidence overlap geographically. Several studies have demonstrated the increment of VL in individuals with acute malaria [29,32,41,42]. Moreover, the risk of HIV infection is higher in areas with high malaria prevalence [43], and malaria has been proposed as an important facilitator for the spread of HIV in sub-Saharan Africa [36,43]. Malaria is a disease caused by protozoa of the genus *Plasmodium*, which are transmitted as sporozoites through bites of infected female *Anopheles *mosquitoes. During this life cycle stage, the sporozoites invade hepatocytes and replicate asexually. Each sporozoite produces thousands of merozoites per infected hepathocyte. This stage is followed by the invasion of mature red blood cells (erythrocytes) by merozoites. The rupture of an infected erythrocyte releases a few dozens of merozoite progeny that are competent to infect new erythrocytes and thus begin a new cycle [44,45].

To introduce malaria parasites into the system, we implemented a model developed earlier [46] and used by several authors [47-50] to study the population dynamics of *X *uninfected red blood cells, *Y *infected red blood cells, *M *free merozoites, and *T_m _*activated immune cells against malaria infection. This model focuses on the erythrocytic cycle of the parasite with an immunological response by the host directed against merozoite and infected red blood cells. The model assumes that the net rate of red blood cells infection is proportional to the density of uninfected red blood cells and the density population of free merozoites (*k_m_MX*). Likewise, the *T *immune cell proliferation is proportional to the density of the *T *cell and free merozoite populations (*λ_m_MT_m_*), and the T cell-mediated killing of free merozoites and infected red blood cells are proportional to the density of the malaria specific immune cells (*μ_n_T_m_M*), ignoring the stimulation of B cells and other type of immune cells (processes that most probably are summarized by the density of the specific T cells). The system is described by the following system of differential equations:

(5) $$\frac{dX}{dt}=\Delta -\mu_{x}X-k_{m}MX$$

(6) $$\frac{dY}{dt}=k_{m}MX-\left(\mu_{1}+\mu_{y}\right)Y-\mu_{c}YT_{m}$$

(7) $$\frac{dM}{dt}=r\mu_{1}Y-k_{m}MX-\left(\mu_{m}+\mu_{h}T_{m}\right)M$$

(8) $$\frac{dT_{m}}{dt}=\varepsilon +\left[\lambda_{y}Y+\lambda_{m}M\right]T_{m}-d_{t}T_{m},$$

where Δ stands for the rate of red blood cell production, *μ_x _*the natural death rate of uninfected red blood cells, *k_m _*the infection rate of red blood cells by merozoites, *μ_1 _*the differentiation rate of merozoites, *μ_y _*natural death rate of infected red blood cells, *μ_c _*clearance rate of infected red blood cells due to the immune system, *μ_m _*natural death rate of free merozoites, *μ_n _*death rate of merozoites by contact with immune cells, *r *merozoite production per infected cell, *ε *rate of immune cell production, *λ_y _*proliferation rate of immune cells in response to infected red blood cells, and *λ_m _*proliferation rate of immune cells in response to merozoites. The parameter values are summarized in Table 1.

### Within-host co-infection model

To link the within-host and between-host model, we developed a so-called nested model that connects the dynamic processes that occur at both scales. These kind of models have been previously used in epidemiology primarily to understand the pathogen evolution [51]. As previously discussed, co-infection activates host immunity, which in turn enhances HIV replication [27]. This phenomenon has been suggested to be a direct consequence of T helper cell (CD4+ T) activation that consequently raises the number of target cells susceptible to virus infection [52]. The increment in the pool of susceptible target cells has been observed in reaction to different agents that induce an immune response such as influenza vaccine [53], tetanus immunization [33,54], and malaria parasites [42,55]. This biological argument was used to link the HIV and malaria models in a single co-infection model, and has also been used by Jones and Perelson [56] to model the effect of vaccination on chronically HIV positive persons and the effect of opportunistic infections on patients treated with antiretroviral therapy [57]. But the within-host co-infection model developed here is the first model to include the dynamics of more than one infection simultaneously to evaluate the effect of a concomitant infection on the dynamics of HIV.

It is important to note, however, that the immune stimulation that promotes HIV replication is multifactorial, and cell proliferation is only one of these mechanisms. For example, malaria infection also stimulates HIV transcription in other ways such as the activation of viral transcription via cytokines [55]. Therefore, the model presented here is a simplification of the effect of co-infection on the virus population and only describes one of the possible mechanisms in which parasites might affect the immune pathway that could alter the replication of the virus.

For the complete model of co-infection, the HIV and malaria models previously described were linked by depicting the mechanism in which malaria parasites stimulates an immune response in terms of activation and proliferation of malaria activated immune cells (*T_m_*), which in turn become available to HIV infection. The model includes a maximum number of immune cells per mL of blood (*T_max_*) [58]; thus, the immune cell population will grow in a logistic fashion. After linking the two models, the dynamic of the HIV-malaria co-infection is described by the following system of differential equations:

(9) $$\frac{dX}{dt}=\Delta -\mu_{x}X-k_{m}MX$$

(11) $$\frac{dY}{dt}=k_{m}MX-\left(\mu_{1}+\mu_{y}\right)Y-\mu_{c}YT_{m}$$

(12) $$\frac{dM}{dt}=r\mu_{1}Y-k_{m}MX-\left(\mu_{m}+\mu_{h}T_{m}\right)M$$

(13) $$\frac{dT_{m}}{dt}=\varepsilon +\left[\lambda_{y}Y+\lambda_{m}M\right]\left[1-\frac{T_{m}+T+T^{*}}{T_{\text{max}}}\right]T_{m}-\left(d_{t}+k_{Tm}V\right)T_{m}$$

(14) $$\frac{dT}{dt}=s+\left[1-\frac{T_{m}+T+T^{*}}{T_{\text{max}}}\right]r_{T}T-\left(d_{t}+k_{v}V\right)T$$

(15) $$\frac{dT^{*}}{dt}=k_{v}VT+k_{Tm}VT_{m}-\delta T^{*}$$

(16) $$\frac{dV}{dt}=N\delta T^{*}-cV.$$

With this model, we estimated the effect of the HIV infection rate (*k_v_*) and production rate (*N*) on the increment of the VL induced by co-infection by conducting several numerical simulations of the model evaluated over the bi-dimensional parameter space generated by the range parameter values of 0.1 × 10^-5 ^to 2 × 10^-5^/day for the HIV infection rate (*k_v_*), and 20 to 300 virions/cell for the HIV production rate (*N*). We first simulated the system with HIV alone (in absence of co-infection), and then we generated several simulations with the complete system (HIV/malaria co-infection). Due to the uncertainty associated with HIV infection rate of immune cells produced by the presence of malaria parasites (*k_Tm_*), we explored four different values for this parameter, which were proportional to the values used for the HIV infection rate (*k_v_*): *k_Tm _*= *k_v_, k_Tm _*= *k_v_*/10, *k_Tm _*= *k_v_*/20, *k_Tm _*= *k_v_*/30. The simulations started with the introduction of HIV alone; when the dynamics of the virus reached a steady state equilibrium (the spVL), parasites were introduced into the system (day *t *= 600). We recorded the VL immediately before the introduction of parasites, which represented the value for the spVL, and the following 30 days after the beginning of the rise of the VL generated by co-infection. The values recorded during these 30 days were then averaged to estimate the increment on the VL generated by co-infection.

To compare spVL and the increment on the VL induced by co-infection, we sampled the parameter space (*k_v_, N*) across several diagonal transects which generated broad combinations of the two parameters. These combinations were then used to calculate the spVL and its corresponding increment on the VL induced by co-infection. This information was then summarized in the geometric per capita growth rate of the virus during co-infection estimated for each combination of parameters.

### Effect of co-infection at the population level

Understanding how concomitant infections could influence between-host transmission and epidemic dynamics represents a major challenge in epidemiology. The scenario is even more complicated when possible viral genetic differences might introduce heterogeneity into the outcome of co-infection. For that reason, using the within-host model described previously, we attempted to evaluate the effect of virus heterogeneity on transient co-infection-induced amplification of the VL at population level.

To estimate the effect of co-infection on the spread of HIV at population level, we implemented a standard deterministic population model constructed by Abu-Raddad and coworkers [36,59]. The model is a deterministic compartmental model that stratifies the population into compartments according to HIV sero-status and stage of HIV infection, and sexual-risk activity group. The model included the three stages of HIV infection, acute, chronic and late, each one with its corresponding probability of transmission per sexual contact estimated from the literature (in the Additional file 1: Table S1). Details of the model are described elsewhere [36,59], and a short description of the model is included in the Additional file 1.

For this model, the functional relationship between VL and the probability of transmission per sexual contact was used as the within-host and between-host interface. The model assumes that the effect of the VL on the infectiousness follows the empirical relationship between VL and transmission probability per sexual contact as observed initially by Quinn and colleagues [23]: $p_{I\left(i\right)\to S\left(j\right)}^{HIV}=2.45^{\text{log}\left(vlH/vlB\right)}p_{}^{HIV}$, where $p_{I\left(i\right)\to S\left(j\right)}^{HIV}$ stands for the probability of transmission per sexual contact for an infected individual from the risk group (*i*) to a susceptible individual from the risk group (*j*), *p^HIV ^*is the baseline probability of transmission per sexual contact in the chronic stage of the infection, *vlH *is the amplified VL, and *vlB *is the baseline VL. The baseline probability of transmission per sexual contact in chronic stage was assumed to be *p^HIV ^*= 0.0008, with a VL of *vlB *= 15 000 virions/mL [23]. Thus, as an example of the calculations performed, at *vlH *= 33 478 virions/mL, the probability of transmission per sexual contact in the chronic stage is $p_{I\left(i\right)\to S\left(j\right)}^{HIV}=2.45^{\text{log}\left(\text{33478}/15000\right)}\times 0.0008=0.00109$. Finally, to account for the potential effect of the VL on the HIV disease progression to AIDS, each log_10 _rise in the VL increases the rate of HIV progression to the latest stage of the infection by twofold [28].

The spVL and its corresponding co-infection induced increment on the VL previously evaluated for each combination of parameters were used to compare the spread of HIV in two different populations: one population free of co-infection, in which the probability of transmission per sexual contact during the chronic stage of the HIV infection was estimated at the spVL, and the corresponding population affected by co-infection, in which the probability of transmission during the chronic stage of the HIV infection was estimated by including the increment on the VL induced by the concomitant infection with malaria. To calculate the probability of transmission in the population affected by co-infection, it was assumed that each individual has, on average, one episode of malaria infection per year. Thus, the mean VL per year was the average of the spVL during 11 months and the increased VL for the month of the co-infection. The model was also evaluated assuming two and four episodes per year.

The system was evaluated at the endemic equilibrium (see the Additional file 1 for details). After the equilibrium solution was found through convergent successive approximations, the total incidence was calculated. Finally, to evaluate the effect of co-infection under different viral replicative capacities, the direct effect of co-infection on the HIV incidence was measured by using population attributable fractions (PAF), which is defined as the proportional reduction in disease risk over a specific time interval that can be achieved by eliminating HIV/malaria co-infection from the population [60,61]. It is calculated as:

(17) $$PAF=\left(1-\frac{IR_{vlsp}}{IR_{Hvl}}\right)\times 100\%,$$

where *IR_vlsp _*is the incidence rate of HIV with the spVL, and *IR_Hvl _*is the HIV incidence rate with the heightened VL in presence of co-infection for each combination of parameters *k_v _*and *N *as defined previously [36]. Due to the absence of consistent data reporting the effect of malaria-related morbidity on sexual behavior [62], we assumed that the episodes of malaria were asymptomatic as a consequence of the partial immunity generated by the consistent exposure to the parasite in areas where malaria is endemic [63,64].

## Results

### Within-host model

**Figure 1:**
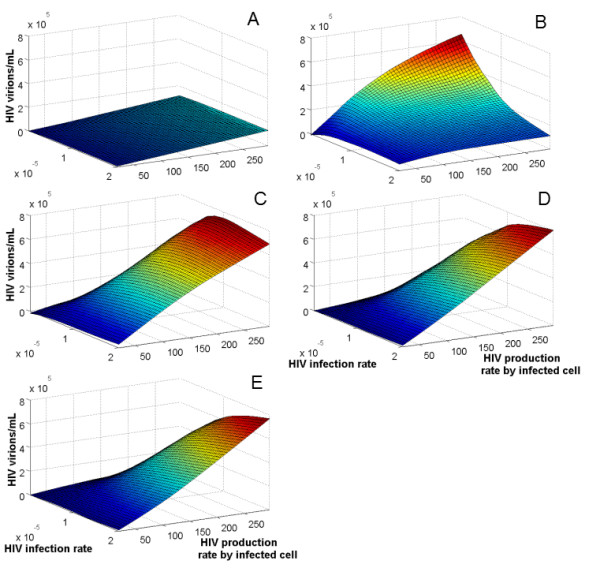
**Increment on VL induced by co-infection as a result of HIV replicative capacity**. The effect of HIV infection rate (*k_v_*) and production rate (*N*) on the increment of the VL generated by co-infection was evaluated using the bidimensional parameter space with range 0.1 × 10^-5 ^to 2 × 10^-5^/day for *k_v_*, and 20 to 300 virions/cell for *N*. In A spVL in absence of co-infection; B *k_Tm _*= *k_v_*; C *k_Tm _*= *k_v_*/10; D *k_Tm _*= *k_v_*/20; and E *k_Tm _*= *k_v_*/30; where *k_Tm _*indicates the HIV infection rate for malaria immune cells.

The graph for the spVL (in absence of co-infection), generated in the parameter space implemented, indicated that the values for the spVL were mainly dependent on the virus replicative capacity (*N*) (Figure 1A). In contrast, the infection rate of immune cells (*k_v_*) had a small effect on the estimated spVL. This result might be related to the limited number of immune cells available to be infected by the virus. Therefore, regardless of the capacity of the virus to infect target cells, the spVL is limited by the number of available cells present in the system. For that reason, the number of virions produced by an infected cell (*N*) is the main parameter that governed the estimated spVL.

**Figure 2:**
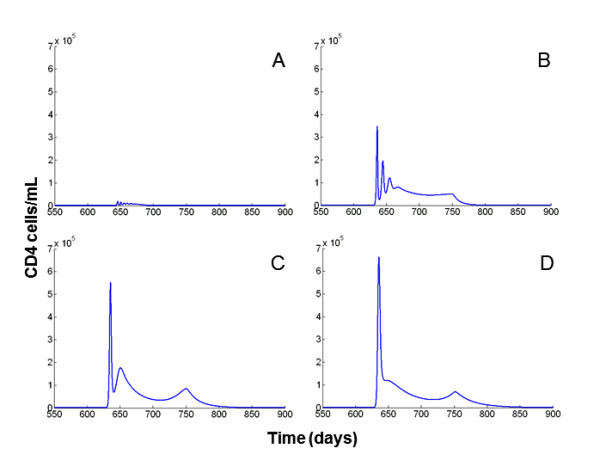
**Time course of the CD4 cells in presence of co-infection with different HIV infection rates of malaria immune cells *k_Tm_***. Figures illustrate the CD4 cell dynamics from day 550 to day 900 keeping *N *and *k_v _*constant: *k_v _*= 1.2 × 10^-5 ^and *N *= 200. For A, *k_Tm _*= *k_v_*, the virus rapidly infects the new target cells impeding the proliferation of the CD4 cells. In B, *k_Tm _*= *k_v_*/10, the effect of the overexploitation of target cells by the virus is still evident. In C and D this effect is diminished, with *k_Tm _*= *k_v_*/20 and *k_Tm _*= *k_v_*/30 respectively.

On the other hand, the results from the complete system (HIV/malaria co-infection) indicated that both parameters, the infection rate of immune cells (*k_v_*) and the production rate by infected cells (*N*), determine the amplification of the VL in presence of co-infection. In general, the within host co-infection model indicated that small values for the two parameters evaluated, the HIV production rate by infected cell (*N*) and the HIV infection rate (*k_v_*), produced small increments on the VL when dual infection with HIV and malaria is present. Conversely, the increment on the VL increased as both parameters increased. Different values for *k_Tm _*(HIV infection rate of immune cells for co-infection), however, generated different patterns. When *k_Tm _*= *k_v_*, the differences on the increment of the VL in response to co-infection were determined by the variation on the HIV production rate per infected cell (*N*) (Figure 1B). The highest VL amplification was produced at a very low HIV infection rate (*k_v _*= 0.1 × 10^-5^) and high HIV production rate per infected cell (*N *= 300). Contrary to expectation, when *k_v _*increases, the increment in the VL generated by co-infection decreases. This behavior is derived from the ability of the virus to infect new target cells activated by malaria parasites. At high *k_v_*, the virus rapidly infects the new target cells impeding the proliferation of these immune cells (Figure 2A). Consequently, this overexploitation of the new target cells by the virus at early stages of co-infection prevented an effective immune response to control malaria. This scenario seems to be unrealistic since individuals infected with HIV are able to have an effective immune cell proliferation in the presence of concomitant infections [65,66]. In addition, the increment on the VL in individuals dually infected with HIV and another pathogen is associated with an increment on the CD4+ T cells [41,42].

On the other hand, when the *k_Tm _*was a fraction of the *k_v_*, the increment of both parameters, the HIV production rate per infected cell and the HIV infection rate, induced larger increments in the VL in response to co-infection. This result suggests that a large *k_Tm _*might affect the availability of the new target cells for infection by the virus by diminishing the potential cell proliferation (Figure 2). When *k_Tm _*= *k_v_*/10, the effect of the overexploitation of target cells by the virus is still evident at high HIV infection rate (starting from *k_v _*= 1 × 10^5^) (Figure 1C). This effect is diluted when *k_Tm _*= *k_v_*/20 (Figure 1D), and the target cell concentration stabilizes with no substantial changes at smaller fractions of *k_v _*(Figure 1E). For that reason, and to include possible differences on the HIV infection rate generated by the availability of new target cells for HIV infection, the remaining results were derived assuming *k_Tm _*= *k_v_*/20.

**Figure 3:**
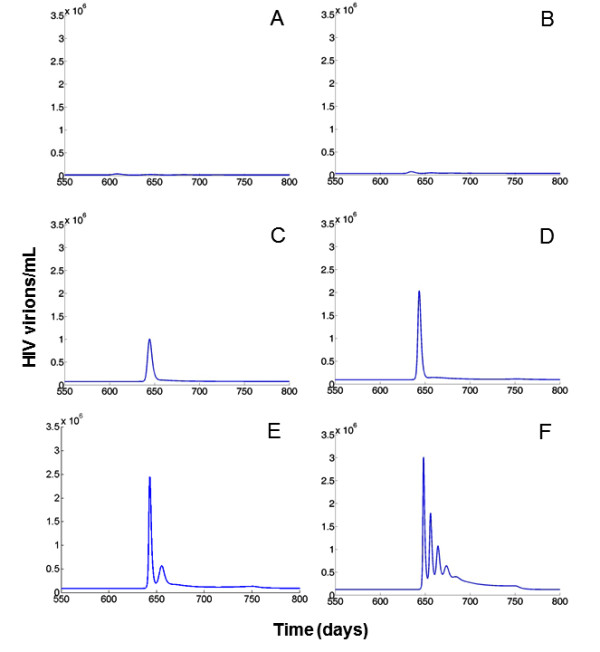
**Time course of the VL in presence of co-infection with different set of parameters *k_v _*and *N***. Figures illustrate the HIV dynamics from day 550 to day 800 for selected set of parameters from Figure 1C. In A, *k_v _*= 0.2 × 10^-5 ^and *N *= 50; B, *k_v _*= 0.5 × 10^-5 ^and *N *= 120; C, *k_v _*= 0.6 × 10^-5 ^and *N *= 170; D, *k_v _*= 0.9 × 10^-5 ^and *N *= 190, E, *k_v _*= 1.2 × 10^-5 ^and *N *= 200; F, *k_v _*= 1.8 × 10^-5 ^and *N *= 280

Figure 3 illustrates the temporal dynamics of the viral load according to the virus parameters used. The combination of parameters generating low spVL were almost unable to produce an increment on the VL when parasites were introduced into the system (Figure 3A, B, and 3C). Conversely, larger spVL associated with more efficient viral replicative capacity generated much larger increments in the VL in response to co-infection (Figure 3D, E, and 3F). After the introduction of parasites, there was a transient increment on virus concentration followed by a peak and successive decay to the original spVL. It is possible to observe a time delay between the introduction of the parasite and the peak of the VL (approximately 45-50 days). It might represent the time it takes for the parasite to increment its population to generate an immunological response required to perturb the VL. These viral dynamics in response to immune stimulations have been observed previously *in vivo *[53,55] and replicated by other mathematical models [56,57].

On average, the VL returned to baseline levels six to eight weeks after the immune stimulation, consistent with other studies in which a large increase in the VL was observed during acute malaria and after influenza vaccine [32,53]. The average increment in the VL generated by co-infection, estimated from the 30 days recorded during the co-infection episode, ranged from 500 to 800 000 virions/mL. This variation is consistent with the ranges estimated from studies designed to evaluate the effect of malaria on the VL of HIV-positive individuals in Africa [32,42].

**Figure 4:**
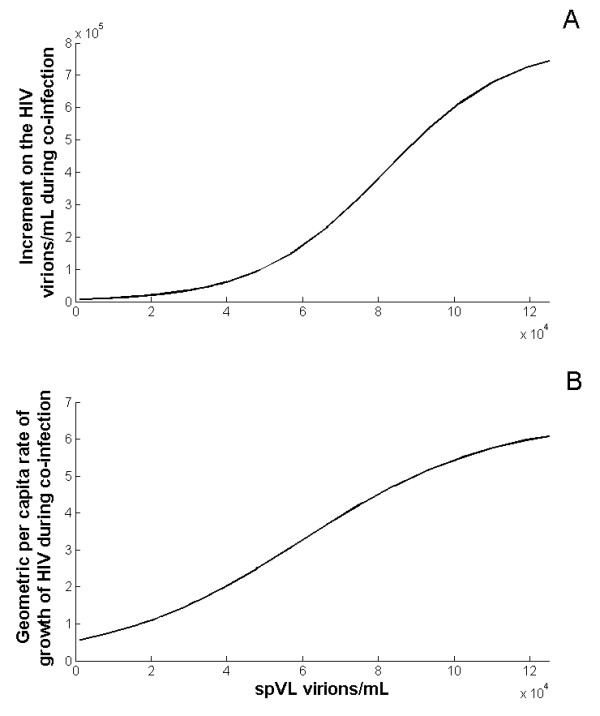
**Correlation between the spVL and the increment on the VL in response to co-infection**. We used different combination of the parameters *k_v _*and *N*, in which both parameters increase. The values used are reported in Table S1 in the Additional file 1. In A, the results suggest that viruses with low replicative capacity conducing to low spVL are generating limited increments on the VL. Conversely, viruses with potent replicative capacity that are able to produce elevated spVL would generate much larger increments on the VL. The pattern follows a logistic fit, which suggests a saturation effect at very high spVL. In B, the geometric per capita rate of growth was estimated for each spVL, and the pattern also shows a saturation fashion.

The positive correlation between the spVL and the increment on the VL induced by co-infection is illustrated in Figure 4A, which was generated with the parameter values reported in Table S2 (see Additional file 1) for default *k_Tm _*= *k_v_*/20. Viral genotypes with poor infection rate of immune cells (*k_v_*) and the production rate by infected cells (*N*), were not able to respond to increments in the activated target cells induced by the presence of concomitant infections. Consequently, the co-infection related increment in the VL generated by these viruses was fairly small. At lower spVL, the relationship between the increment on the virus concentration and the spVL is exponential, suggesting that viral genotypes with potent replicative capacity, and thus elevated spVL, were associated with large co-infection induced increments in the VL. The pattern followed a logistic function, suggesting a saturation effect at very high spVL (Figure 4A). Additionally, the high HIV per capita growth rate during co-infection observed at large spVL might reflect the high replication capacity of these viruses that were able to effectively respond to increments in the target cell population (Figure 4B).

### Between-host model

**Figure 5:**
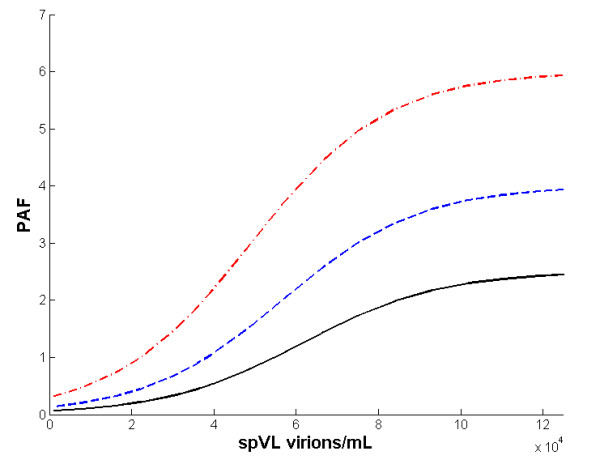
**Correlation between the spVL and the PAF**. Using combinations of the parameters *k_v _*and *N *(see Table S2 in the Additional file 1), the between-host model indicated that co-infection would have a negligible impact in populations where the circulating virus has low replicative capacity reflected in low spVL. Conversely, the impact of co-infection would increase as the spVL of the population increases, until the effect tends to stabilize at spVL around 1 × 10^5^. Black solid line indicates the PAF estimated from one episode of malaria; blue dashed line, two episodes; red dashed-dotted line, four episodes.

The functional relationship between the viral load and the probability of transmission per sexual contact was used to link the viral replicative capacity and the effect of co-infection at the population level. As expected, and in accordance with the immunological model, the PAF analysis indicated that co-infection would have a negligible impact in populations where the circulating virus has low replicative capacity, reflected in low spVL. In contrast, the impact of co-infection would increase as the spVL of the population increased, and this effect levels off at around spVL 1 × 10^5 ^(Figure 5). This result indicates that viruses with high replicative capacity that are able to maintain elevated spVL would be those that have impact on the spread of the infection in terms of new HIV infections directly caused by co-infection. This result was consistent regardless of the average number of co-infection episodes per year. In addition, the number of co-infection episodes has almost no impact on the PAF at low spVL; even four episodes were not able to produce a PAF larger than 1% when the spVL is lower than 2 × 10^4 ^.

## Discussion

In this work, we explored factors that could introduce variation in the effect of co-infection on HIV dynamics at individual and population levels. We postulated that if the HIV replication capacity was an important determinant of the spVL, it would also determine the effect of co-infection on the dynamics of the virus. Here, using a previously described mathematical model to represent the single dynamics of HIV at host level [38,39], it was determined that the variation in the viral replication capacity, described primarily by the HIV production by infected cell (*N*), generated different viral load set-points. Moreover, the influence of the virus replicative capacity on the increment in VL generated by co-infection was evaluated using a within-host mathematical model of HIV/malaria co-infection which examined the effect of variation in viral infection rate of immune cells (*k_v_*) and the production rate by infected cells (*N*). The model indicated that the viral replication capacity regulated by these parameters would strongly influence the induced increment of the VL when a concomitant infection is introduced in the system. In contrast to the spVL, in which the infection rate plays a minor role and is primarily determined by the production rate per infected cell, the new available target cells generated by the presence of a concomitant pathogen is a suitable condition for virus genotypes with higher infection rate of immune cells.

In addition, our model suggested the existence of a differential HIV infectivity rate for the new target cells generated by co-infection. Infection agents such as *Plasmodium *induce localized immune response occurring in specific organs such as the spleen [66], which produce a decrease in peripheral T cells as consequence of a sequestration of these cells to specific lymphoid organs [65]. These characteristics might generate a compartmentalization of the system in which not all new target cells would be available to the virus. Consequently, the HIV infection rate for the new target cells generated by co-infection (*k_Tm _*in our model) might be a fraction of the HIV infection rate for non-specific immune cells (*k*_v_), as suggested by the model.

Low virus replicative capacity generated modest increments on the VL in response to co-infection. Regardless of the co-infection induced proliferation of target cells, the low replicative capacity prevented an effective viral response to the newly available activated target cells. Conversely, viruses with more efficient capacity to infect target cells and replicate were able to produce much stronger increments on the VL when a larger population of target cells was available. Despite the introduction of new target cells to the system, the effect of co-infection could primarily depend on the ability of the virus to efficiently exploit the temporal rise of the target cell population. This, in consequence, would generate a considerable increment on the VL when the immune system of the host is stimulated by the presence of a concomitant pathogen. This result suggests that viral factors could play an important role not only in determining the spVL but also in driving several virus-related processes such as the increment of the VL induced by co-infections.

Our results would have important implications for the identification of highly infectious individuals. HIV-positive persons that sustain high spVL might therefore be able to produce large increments of virus concentration in response to immune stimulations and consequently become much more infectious individuals, compared to those maintaining low spVL, even in the presence of the same concomitant pathogen. This argument was then evaluated at the population level by linking the results from the within-host model with a population model using the functional relationship between viral load and probability of HIV transmission per sexual contact. We generated several virtual populations with different viral replicative capacities that sustained different mean spVL in which the direct effect of co-infection on the incidence of HIV was then evaluated. The results from these simulations were consistent with the results from the host level model and raise the possibility that co-infection might play a substantial role in the spread of HIV in populations where the circulating viruses maintain elevated spVL. Conversely, co-infection would not be an important driver of the epidemic in populations with low spVL.

This study is a theoretical exercise aimed to explore further implications of the variation of viral genetic factors on the natural history of HIV. To achieve this goal, we developed a within-host co-infection model based on two well-known and extensively used mathematical models describing the within-host dynamics of HIV and malaria infections. These two models were linked by the immune response induced by the concomitant infection with *Plasmodium *that increased the target cell population for HIV infection. This mechanism, however, is only one of the several mechanisms in which the invasion of another pathogen stimulates the replication of HIV [41,53,67,68]. The interaction between HIV and concomitant pathogens appears to be multi-factorial at cellular and immunological levels [67]. For example, the transcriptional signaling used by lymphocyte cells to regulate cell functioning is also used by HIV to regulate virus production. During co-infection, cytokines (small proteins secreted by specific cells of the immune system used for local signals between cells during response to infection [69]) might enhance their susceptibility to HIV infection and stimulate production of viruses for weeks to months without significant cytopathic effects [70,71].

Moreover, other infections might have additional mechanisms in which they could affect the transmission of HIV. For example, the genital reactivations of HSV-2 generate local immune activations in genital ulcerations that might increment HIV shedding in the genital tracks [72]. This STI infection not only affects the risk of transmission but also increases the risk of acquisition in HIV-negative individuals, a characteristic that considerably affects the mode in which HSV-2 influences the pattern of HIV transmission at the population level [36,59]. The differences in which other infections, besides malaria, interact with HIV are not considered in this study, as they may affect the results observed at the population level.

We emphasize that this work relies entirely on a hypothetical relationship between viral genotypic factors and the VL. As noted previously, regardless of the evidence about the relationship between viral genetic factors and the VL [15-17], the role of virus genotype on the natural history of HIV infection is controversial [73,74], and the evidence about differences in VL among populations is limited and very speculative [3,75]. Therefore, our study indicates the importance of conducting such studies that focus on estimating the VL to elucidate possible differences at population level. Such studies would help us better understand the extensive variation on the HIV epidemic.

Furthermore, our model does not include an evolutionary response of HIV. The virus might evolve at the two scales studied; at between-host level, the virus evolves rapidly to evade the host immune system [76]. This selection pressure favors the increment of the virus replicative capacity in the course of the infection [19]. On the other hand, at between-host level some theoretical studies have suggested that the trade-off generated between the transmission and progression of the infection implies and evolutionary stable strategy that favors virus strains with intermediate virulence [77,78]. These selection forces acting on both scales might have some important implications on the transmission efficiency of the virus that might alter the results discussed in this study. Future work would include the potential impact of these forces acting on the virus replicative capacity and the interactions with a concomitant pathogen.

Regardless of the limitations previously discussed, the model presented here highlights the possibility that viral factors might have an important impact on the role of co-infection on the spread of HIV. Therefore, understanding their variations would be a key element in the design of control interventions to prevent HIV transmission. To pursue this goal, however, it would be necessary to evaluate the results from the theoretical model proposed here with experimental data. These kinds of studies should be conducted in HIV-positive individuals in the chronic stage of the infection with similar CD4+ T cell count, and whose spVL have being well established. Their immune systems could be challenged with the application of previously used antigen immune stimulants such as tetanus toxoid vaccine [33] or influenza vaccine [34,52]. The VL should then be measured frequently (preferably daily) over a considerable period of time (at least one month), to accurately delineate the dynamics of the virus during co-infection.

As suggested by the model, biological differences could alter the effect of co-infection and underscore the importance of identifying these factors for the implementation of effective control interventions focused on co-infection. The effect of co-infection might not be the same among individuals and populations, and control strategies will not necessarily have the same impact in each population. Understanding the role of viral genetic factors on co-infection becomes an important element for developing accurate and effective recommendations for population-level control strategies.

## Abbreviations

spVL: HIV set point viral load; VL: HIV plasma viral load; STI: Sexually transmitted infections; HSV-2: Herpes simplex virus type 2; PAF: Population attributable fraction.

## Competing interests

The authors declare that they have no competing interests.

## Authors' contributions

DFC wrote the draft manuscript and collaborated on project conception, model design, and simulation programming. GGR collaborated on project conception, model design and helped in writing the manuscript.

## Supplementary Material

Additional file 1 **Table S1**. A single PDF file. 9 pages that include supplementary methods. The supplementary tables are embedded in the file [36,59,83-90]. Click here for file
