# Supplementary material for: Variable effect of co-infection on the HIV infectivity: Within-host dynamics and epidemiological significance
Source: Theor Biol Med Model. 2012 Mar 19;9:9. doi: 10.1186/1742-4682-9-9 (PMC3337224; doi:10.1186/1742-4682-9-9)
Supplement: Additional file 1 — Table S1. A single PDF file. 9 pages that include supplementary methods. The supplementary tables are embedded in the file [36,59,83-90]. [file 1742-4682-9-9-S1.PDF]

Variable effect of co-infection on the HIV infectivity: Within-host dynamics and epidemiological significance

Diego F Cuadros[1,2*]
[*]Corresponding author
Email: dfc2002@qatar-med.cornell.edu

Gisela García-Ramos[1]
Email: ggarc0@uky.edu

[1] Department of Biology, University of Kentucky, Lexington, KY, USA

[2] Infectious Disease Epidemiology Group, Weill Cornell Medical College, Doha, Qatar

Additional file 1 - Supplementary material

Between-host mathematical model

To evaluate the effect of co-infection at the population level, we used a standard deterministic population model constructed by Abu-Raddad and coworkers [36, 59]. The model is a deterministic compartment model, which stratifies the population into compartments according to HIV sero-status and stage of HIV infection, and sexual-risk activity group,

$$\frac{dS(i)}{dt} = \mu_p N_0(i) - \mu_p S(i) - \Lambda_i S(i) \qquad (1)$$

$$\frac{dI_1(i)}{dt} = \Lambda_i S(i) - \mu_p I_1(i) - \omega_1 I_1(i) \qquad (2)$$

$$\frac{dI_2(i)}{dt} = \omega_1 I_1(i) - \mu_p I_2(i) - \omega_2 I_2(i) \qquad (3)$$

$$\frac{dI_3(i)}{dt} = \omega_2 I_2(i) - \mu_p I_3(i) - \omega_3 I_3(i) \quad . \qquad (4)$$

The index i stands for an i-sexual risk population where $i=1,2,3,4$ represent the low, low to intermediate, intermediate to high, and high risk groups, respectively. $S(i)$ is the susceptible population, and $I_\alpha(i)$ is the HIV infected populations. The index α indicates the stage of HIV pathogenesis, where $\alpha=1,2,3$ stand for acute, chronic, and late stages respectively, each one with a different probability of transmission per coital act [36]. $N_0(i)$ is the initial population size of each i-risk group.

The progression of HIV is described by ω_1, the rate of progression from acute to chronic stage, ω_2, the rate from chronic to late stage, and ω_3, the rate of HIV/AIDS disease mortality, and μ_p is the death rate. The rates Λ_i are the HIV forces of infection experienced by the susceptible populations given by:

$$\Lambda_i = \rho_{S(i)} \times \sum_{j=1,2,3,4} \left(\sum_{\alpha'=1,2,3} t_{I_{\alpha'}(j)\to S(i)} \Psi(i,j) \frac{\rho_{I_{\alpha'}(j)} I_{\alpha'}(j)}{\rho_{S(j)} S(j) + \sum_{\alpha''=1,2,3} \rho_{I_{\alpha''}(j)} I_{\alpha''}(j)} \right) \quad (5)$$

Here, $\rho_{X(i)}$ describes the new sexual partner acquisition rate for each population $X(i)$. The expression $\Psi(i,j)$ represents the mixing among the four risk groups. It estimates the probability that an individual in risk group i would choose a partner in risk group j [36] and is described by:

$$\Psi(i,j) = e\delta_{i,j} + (1-e) \frac{\rho_{s(j)} S(j) \sum_{\alpha'=1,2,3} \rho_{I_{\alpha'}(j)} I_{\alpha'}(j)}{\sum_{k=1,2,3,4} \left(\rho_{s(k)} S(k) + \sum_{\alpha'=1,2,3} \rho_{I_{\alpha'}(k)} I_{\alpha'}(k) \right)}. \quad (6)$$

Here, $\delta_{i,j}$ is the identity matrix and the parameter $e \in [0,1]$ measures the degree of assortativeness in the mixing. For example, at the extreme $e = 0$, the mixing in the population is fully proportional, while at the other extreme $e = 1$, individuals mix in assortative fashion exclusively.

The parameter t_{I_α} represents the HIV transmission probability per partnership in a partnership between a member of the susceptible population $S(j)$ and a member of the HIV infected population $I_\alpha(i)$. This value is calculated using the binomial model

$$t_{I_\alpha} = 1 - (1 - p^{HIV}_{I_\alpha(i)\to S(j)})^{n_{I_\alpha(i)\leftrightarrow S(j)}\tau_{I_\alpha(i)\leftrightarrow S(j)}}, \qquad (7)$$

which includes the HIV transmission probability per sexual contact per HIV stage ($p^{HIV}_{I_\alpha(i)\to S(j)}$), the frequency of sexual contacts per HIV stage in this partnership ($n_{I_\alpha(i)\leftrightarrow S(j)}$), and the duration of the partnership ($\tau_{I_\alpha(i)\leftrightarrow S(j)}$). Assumptions for parameter values are summarized on Table S1. We focused on the study of the endemic equilibrium solutions of the system, which yields the following expressions for the population variables in terms of the force of infection and other parameters:

$$\overline{S_i} = \frac{\mu_p N_{0,i}}{\mu_p + \Lambda_i} \qquad (8)$$

$$\overline{I_{i,1}} = \frac{\mu_p N_{0,i}}{\mu_p + \omega_1}\left[\frac{\Lambda_i}{\mu_p + \Lambda_i}\right] \qquad (9)$$

$$\overline{I_{i,2}} = \frac{\mu_p \omega_1 N_{0,i}}{(\mu_p + \omega_1)(\mu_p + \omega_2)}\left[\frac{\Lambda_i}{\mu_p + \Lambda_i}\right] \qquad (10)$$

$$\overline{I_{i,3}} = \frac{\mu_p \omega_1 \omega_2 N_{0,i}}{(\mu_p + \omega_1)(\mu_p + \omega_2)(\mu_p + \omega_3)}\left[\frac{\Lambda_i}{\mu_p + \Lambda_i}\right]. \qquad (11)$$

The equilibrium solution was found through convergent successive approximations. Once the force of infection per risk group was determined, we calculated the total incidence using the different values for the $p^{HIV}_{I(i)\to S(j)}$ with and without co-infection.

Table S1. Co-infection between-host model parameter values

Parameter	Value	Reference
HIV transmission probability per coital act per stage of infection (p^{HIV}_{α})		
Acute stage	0.036	[83, 85-86]
Chronic stage	0.0008*	[83]
Late stage	0.0042	[83]
Duration of each HIV stage ($1/\omega_{\alpha}$)		
Acute stage	49 days	[83, 86]
Chronic stage	9.0 years*	[87]
Late stage	2.0 years	[83]
Frequency of coital acts per HIV stage ($n_{I_{\alpha}(i)\to S(j)}$)		
Acute stage	10.6/month	[83]
Chronic stage	11.0/month	[83]
Late stage	7.1/month	[83]
Fraction of the initial population size in each risk group		
Low risk	65%	[88]
Low to intermediate risk	23%	[88]
Low to high risk	9.5%	[88]
High risk	2.5%	[89]
Net sexual partner acquisition rate ($\rho_{X(i)}$)		
Low risk	0.095 partners/year	[36]
Low to intermediate risk	1.30 partners/year	[36]
Low to high risk	5.46 partners/year	[36]

High risk	35.78 partners/year	[36]
Duration of sexual partnership ($\tau_{Y_\alpha(i)\leftrightarrow S(j)}$)		
Low risk with low risk	48 months	[36]
Low risk with low to intermediate risk	36 months	[36]
Low risk with intermediate to high risk	24 months	[36]
Low risk with high risk	12 months	[36]
Low to intermediate risk with low to intermediate risk	24 months	[36]
Low to intermediate risk with intermediate to high risk	12 months	[36]
Low to intermediate risk with high risk	6 months	[36]
Intermediate to high risk with intermediate to high risk	6 months	[36]
Intermediate to high risk with high risk	1 months	[36]
High risk with high risk	1 week	[36]
Duration of sexual lifespan ($1/\mu_p$)	35 years	[36]
Degree of assortativenes (e)	0.224	[59, 90]

*Baseline values that varied according to VL

Table S2. Parameter values used to generate Figure 4

k	N	spVL	Heightened VL
**1.5E-06**	20	1200	6365
**1.8E-06**	30	1300	6403
**1.8E-06**	50	7800	9421
**2.2E-06**	50	16182	15459
**1.8E-06**	60	17300	16509
**2.2E-06**	60	20800	20269
**3.2E-06**	60	22000	21741
**1.8E-06**	80	22900	22913
**2.2E-06**	80	29227	33056
**3.2E-06**	80	30000	34557
**3.9E-06**	80	31700	38091
**2.2E-06**	90	32200	39195
**3.2E-06**	90	34600	44924
**3.9E-06**	90	36000	48622
**4.9E-06**	90	36500	50010
**3.2E-06**	96	37000	51435
**3.9E-06**	96	38600	56253
**4.9E-06**	96	39200	58165
**5.9E-06**	96	39700	59804
**3.9E-06**	116	39900	60472
**4.9E-06**	116	47800	92884
**5.9E-06**	116	48200	94873
**7.0E-06**	116	48700	97410
**4.9E-06**	136	49000	98961
**5.9E-06**	136	57000	148603
**7.0E-06**	136	57500	152267
**9.0E-06**	136	57700	153752
**5.9E-06**	156	58000	156000
**7.0E-06**	156	66100	226041

**9.0E-06**	156	66400	228972
**7.0E-06**	176	66700	231925
**9.0E-06**	176	75000	321465
**12E-06**	176	75500	327250
**14E-06**	176	75600	328411
**9.0E-06**	196	75700	329574
**12E-06**	196	84100	429578
**14E-06**	196	84200	430771
**12E-06**	216	84400	433156
**16E-06**	216	93200	533513
**18E-06**	216	93300	534579
**14E-06**	236	93400	535642
**16E-06**	236	101700	615411
**18E-06**	276	119200	724007
**20E-06**	296	128200	753888

Table S3. Parameter values used to generate Figure 5

k	N	spVL	PAF 1 episode*	PAF 2 episodes*	PAF 4 episodes*
**1.5E-06**	20	1200	0.065409	0.138332	0.321198
**1.8E-06**	30	1300	0.065792	0.139136	0.323027
**1.8E-06**	50	7800	0.095969	0.202147	0.465159
**2.2E-06**	50	16182	0.154805	0.323571	0.732068
**1.8E-06**	60	17300	0.164837	0.34409	0.776289
**2.2E-06**	60	20800	0.20027	0.416131	0.929586
**3.2E-06**	60	22000	0.213942	0.443751	0.987562
**1.8E-06**	80	22900	0.224745	0.465507	1.032923
**2.2E-06**	80	29227	0.315424	0.645747	1.398675
**3.2E-06**	80	30000	0.358639	0.730181	1.564078
**3.9E-06**	80	31700	0.367954	0.748259	1.599017
**2.2E-06**	90	32200	0.415473	0.83982	1.773475
**3.2E-06**	90	34600	0.415473	0.83982	1.773475
**3.9E-06**	90	36000	0.445394	0.896911	1.880184
**4.9E-06**	90	36500	0.456482	0.917957	1.919129
**3.2E-06**	96	37000	0.467782	0.939348	1.958497
**3.9E-06**	96	38600	0.505388	1.010095	2.087187
**4.9E-06**	96	39200	0.520059	1.037517	2.136453
**5.9E-06**	96	39700	0.532524	1.060735	2.1779
**3.9E-06**	116	39900	0.53757	1.070115	2.194575
**4.9E-06**	116	47800	0.763943	1.479086	2.885397
**5.9E-06**	116	48200	0.776734	1.501527	2.92136
**7.0E-06**	116	48700	0.792886	1.529767	2.966343
**4.9E-06**	136	49000	0.802664	1.546807	2.993342
**5.9E-06**	136	57000	1.082956	2.018802	3.700195
**7.0E-06**	136	57500	1.101406	2.048793	3.742592
**9.0E-06**	136	57700	1.108805	2.060784	3.759464
**5.9E-06**	156	58000	1.119923	2.078762	3.784675
**7.0E-06**	156	66100	1.4221	2.550252	4.411659

**9.0E-06**	156	66400	1.433123	2.566848	4.432592
**7.0E-06**	176	66700	1.444117	2.58336	4.453345
**9.0E-06**	176	75000	1.730871	3.000143	4.954293
**12E-06**	176	75500	1.746753	3.022471	4.979944
**14E-06**	176	75600	1.749907	3.026896	4.985014
**9.0E-06**	196	75700	1.753053	3.031308	4.990063
**12E-06**	196	84100	1.988228	3.352749	5.346329
**14E-06**	196	84200	1.990666	3.355998	5.349815
**12E-06**	216	84400	1.995515	3.362455	5.35674
**16E-06**	216	93200	2.175611	3.597682	5.603163
**18E-06**	216	93300	2.177301	3.599848	5.605381
**14E-06**	236	93400	2.178983	3.602004	5.607587
**16E-06**	236	101700	2.294556	3.748296	5.755234
**18E-06**	276	119200	2.42405	3.908163	5.912014
**20E-06**	296	128200	2.455172	3.945964	5.94841

*Episodes of malaria per year
